# Effect of the Partners in Play Intervention on Parents’ Autonomy-Supportive Guiding Behaviour and Children’s Self-Regulation

**DOI:** 10.3390/brainsci14090924

**Published:** 2024-09-16

**Authors:** Natalie Day, Fred Paas, Lisa Kervin, Sahar Bokosmaty, Steven J. Howard

**Affiliations:** 1 Early Start, University of Wollongong, Wollongong 2522, Australia; dayn@uow.edu.au (N.D.); paas@essb.eur.nl (F.P.); lkervin@uow.edu.au (L.K.); 2 Education and Child Studies, Department of Psychology, Erasmus University Rotterdam, 3062 PA Rotterdam, The Netherlands; 3 School of Education, University of Wollongong, Wollongong 2522, Australia; saharb@uow.edu.au

**Keywords:** self-regulation, play, intervention, parents, early childhood, pre-school

## Abstract

Compelling evidence supports the foundational importance of early self-regulation (SR). It also supports parents in the home environment as having the foremost influence on early development. Yet, prevailing approaches to support early SR growth have tended to leverage early education and clinical settings. Partners in Play (PiP) was developed as a sustainable approach for parents to learn how and when to support children through experiences of self-regulation challenges in the home learning environment. This study reports the first randomised control trial evaluation of the PiP program, with 21 parent–child dyads (consisting of twelve girl–mother dyads, eight boy–mother dyads, and one boy–father dyad; mean child age = 4.12 years, SD = 0.65). Dyads were randomised to a PiP intervention group (*n* = 10), which entailed four online parent information sessions and four out-of-home guided practice dyadic play sessions across 8 weeks, or an active control group (*n* = 11). The primary outcome was parent autonomy support, and the secondary outcome was child SR. Results indicated a significant increase in parents’ use of autonomy support and a non-significant but moderate-sized effect on child SR. This innovative proof-of-concept program and evaluation provides a roadmap for effecting change in parental support during children’s play, to the prospective benefit of important abilities such as child SR. Analyses show promise for a parent-based model toward parent behaviour change and child SR improvement.

## 1. Introduction

### 1.1. Background

The way that self-regulation (SR) has been conceptualised positions it as a central mechanism in how we exert volition and control under complex conditions of competing demands and contrary distractions and impulses [1]. Foundational in early life [2], SR predicts both short- and long-term outcomes across the lifespan [3,4]. For instance, early SR is positively associated with academic attainment, social competency, mental well-being, and school engagement, and it is negatively associated with anti-social behaviours such as criminal activity, substance abuse, and unemployment. SR undergoes a period of rapid development between the ages of 3 and 7 years, aligned with significant development and change in the pre-frontal cortex across this period [5,6]. The non-linear way in which this occurs, however, results in different growth trajectories influenced both by genetic predisposition and environment/experience, and thus varies by child, family, and context [2]. Montroy et al. [2] found that 20% of children who showed few gains in pre-school continued to fall behind their peers in SR and, only by age 7, demonstrated SR capabilities that their peers had demonstrated at 4-years old. This gap has also been shown to persist into formal schooling, at least until Grade 6 (11–12 years) [7]. These findings, and evidence of the malleable nature of SR [4], have been the impetus for a body of research into how to support SR growth and mitigate lower-growth trajectories.

While there has been a recent surge in efforts to support SR, there is little consistency in their approach, characteristics, or design (for reviews, see [8,9,10]). These reviews highlight a paucity of parent intervention research [8], despite compelling longitudinal evidence pointing to the home learning environment (HLE) as one of the (if not the) most influential factors on a child’s development [11,12,13,14,15]. In the current research, we intended to respond to this gap by designing, implementing, and evaluating a program to support parents to foster their pre-school-aged children’s self-regulation. This current study adopted a randomised controlled trial design to evaluate, for the first time, the effectiveness of the Partners in Play (PiP) intervention. PiP aims to support children’s SR via changes in parent autonomy support during self-regulation challenge in play. To achieve this, PiP comprises parent information sessions, dyadic parent–child play group workshops with bespoke coaching from an observing facilitator, and at-home play. Accordingly, the primary outcome for PiP is parent autonomy-supportive behaviour during children’s play and, thereby, children’s SR (a secondary outcome). 

### 1.2. Theoretical Basis of SR

SR is simultaneously conceived as a foundational ability, a complex process, and a desirable outcome. As such, there is diversity in its operationalisation and terminologies to define SR [16]. One prominent model, which informs our conception of SR for this research, is Control Theory—in particular, Carver and Scheier’s Feedback Loop Model [17]. Three key elements of SR are proposed in this model: standards (self-selected ideals/goals); monitoring (comparison of the current state to the desired ideals/goals); and operate (the action that is required to close the discrepancy between the current state and the ideals/goals) [18,19]. In addition, Baumeister and Heatherton [20] proposed a capacity component: to move forward toward goal attainment when confronted with contrary impulses and distractions. Commonly considered as enabled by executive functions (EFs) [21], these higher-order cognitive processes are responsible for top-down regulation of our behaviour [22], including flexible adaptation to changing environments, inhibition of impulses and resisting distraction, and the ability to concurrently hold in mind and mentally work with information [23]. While EFs are necessary for SR, according to this model, they alone are not sufficient—that is, they fail to account for the volitional element of SR as evidenced by delay of gratification studies [24,25,26,27]. Performance on these tasks is attributed not only to cognitive (EF) processes but also to the value of the goal and motivations driving goal (non-)completion, as captured in Baumeister and Heatherton’s model [20]. 

While EFs support SR in a top-down direction, context-dependent (bottom-up) factors can also influence available self-regulatory capacity and its manifestation. For instance, highlighting the instability of moment-to-moment SR [28], research has shown that a child’s ability to self-regulate is negatively influenced by factors such as critical parenting behaviours consisting of coercion and criticism [29], stress, tiredness [30], and a lack of enriching activities in the HLE [29]. This bi-directionality of influence impacting SR [31] identifies plausible targets for SR-promoting programs: on one hand, promoting constituent abilities/strategies underpinning self-regulatory control (goal setting, motivation, and capacity) while at the same time considering children’s reactivity to in situ SR challenges. Yet, while this model of SR provides an understanding into the dynamic factors impacting processes and outcomes of children’s attempts at SR, it does not offer insight into how to action these mechanisms of change. There remains little consensus on intervention characteristics shown to be conducive to SR growth.

### 1.3. Mechanisms of SR Change 

Primarily a theory of motivation, Self-Determination Theory (SDT) [32] offers a framework for how critical aspects of SR might be influenced. Specifically, SDT posits a positive association between the degree of fulfilment of basic psychological needs and the intrinsic motivation toward regulating behaviour. The three needs are autonomy, competence, and relatedness [33]. Autonomy refers to a perceived internal locus of control for actions [34]. It is nurtured through a provision of choice and acknowledgement, recognition, and validation of feelings, and it is undermined by externally imposed goals, negative judgement, and insensitivity [33]. Autonomy impacts the extent to which behaviours are enacted with a sense of volition (the ‘self’ aspect of self-regulation; [32]), rather than heteronomy (control by external actions or internal compulsions, as in other- or co-regulation; [35]). The latter often yields compliant behaviour. Competence is cultivated when experiencing success after (appropriate) challenge and is linked with mastery. Fulfilment of competence can be achieved with positive encouragement and feedback and avoiding negative self-appraisal. Relatedness pertains to a perceived sense of genuine care from others. It is nurtured through a lack of criticism, leading to feelings of belonging and security, for example. 

A recent review of pre-school SR interventions [10] promisingly showed that programs that embedded aspects of SDT were successful in generating SR growth, albeit to differing extents depending on the nature and scope of integration. For instance, programs with only a relatedness element of SDT showed significant but typically small SR effects. Programs that featured the competence element of SDT, using encouragement and feedback, were associated with the largest SR change. This was also the case when these programs included elements of autonomy support (e.g., via shared control, child-led activities, and choice). This is consistent with evidence that the experience of challenge (which threatens regulatory behaviour) that is successfully navigated is necessary for cognitive growth [36]. This review, which largely included studies that used task-based measures to capture SR change (given biases inherent in self-reported outcome measures [37]), thus provides insight and evidence into the potential of SDT as a guide for how to achieve change in crucial aspects of SR. 

The scoping review also highlighted some pragmatic and implementation characteristics that more often featured amongst programs that generated SR change. Unexpectedly, given the suggestions in the literature [36,38], this review did not find clear impacts of dosage and duration on SR change across interventions. Muir et al. [39] found that particular intervention practices were more important than dosage. Opportunities to practice skills, accompanied by feedback, were more evident amongst effective studies, which aligns with the competence component of SDT. Coaching was also a component that more often featured in effective SR programs, satisfying parents’ feelings of competence by goal setting and challenge [39]. Coaching offers a tailored approach to behaviour change along with specific feedback [40]. 

While the authors’ scoping review highlighted the conditions under which to optimise regulation, how adults contribute to dysregulation resolution as co-regulators at the micro-level is worthy of comment. Autonomy-supportive guidance from adult caregivers supports children’s SR development [41,42,43,44,45] and, thus, offers a way for how parents can support child SR—encompassing both autonomy and competence elements of SDT. It is proposed that when parents use autonomy-supporting guiding strategies, they set the tone for children to experience agency, as defined by Sen [46]—“agency is not just choosing something or expressing something, but being able to influence and make decisions so that capabilities can expand” (p6). 

### 1.4. Play as a Vehicle of SR Development 

Sociocultural theories advocate play as the most significant learning activity of early childhood [47], a time in which children become capable of regulated, intentional behaviour and use their “cultural tools to transform their cognitive process, such as perception, attention, memory and thinking” [48], p. 373). Through the act of play, children develop the quality of their thinking, problem solving, and creativity [49]. In symbolic pretend play, for example, a child experiences an intra-relationship between an object and the role it has, mediated by the meaning the child places on the object [50]. In this way, a stick does not stay a stick but becomes a tool or a wand dependent upon the play and social environment that the child is a part of. For these various reasons, play has been shown to have positive effects on social development [51], emotional regulation [52], language development [53], self-regulation [50], and attachment [54].

While play is defined in different ways, one prominent conceptualisation holds that play can be expressed as unfolding across a continuum on which child autonomy varies [55], from free play depicted by full child-autonomy (e.g., pretend play, including role play and symbolic play [56]) to direct adult instruction in the absence of child autonomy. Play that is driven by a child’s interests is believed to elicit intrinsic motivation to regulate behaviour (according to SDT [32]). It is not so much play types that promote (or not) SR, but perhaps also the tools of play, which include the environment that affect the level of SR challenge encountered and play partners who are able to offer support as co-regulators in moments of SR challenge. 

Aligned with Vygotsky [53] and other sociocultural theorists [57], the term *social situational development* (which describes the importance of the child’s interactions with others and in the family context) pertains that development is dependent on the parent–child relationship. As such, we invite a wider holistic view that looks beyond the outcome of play to look at the quality of the social interaction, the how of the play (rather than the what) between a parent and child to contribute toward the reciprocal development toward autonomy and agency expression, and opportunities for cognitive development. This idea embraces the bi-directional model of SR [31] and, notably, the ‘bottom-up’ influences on a child’s ability to regulate their behaviours, as a result of their motivation to do so. By supporting children’s autonomy during experiences of SR challenge in play, parents can ‘carve out’ a space in which children can experience agency and autonomy. 

### 1.5. Current Study 

There is a dearth of evidence-informed programs that leverage the HLE to provide parents with the knowledge of how to integrate effective responses into their play interactions with their children. Also, where these exist, there are few clear theories of change that inform their design and implementation. The current study designed and implemented a parent program based on a model of SR, the top-down and bottom-up influences on SR, and SDT principles suggesting how to effect change in critical SR components. 

In the current study, we thus conducted a randomised control trial (RCT) of Partners in Play (PiP), a parent-based intervention that capitalises on the play-context that coached parents toward sustainable SR support. The goal of this intervention was twofold: the first was to provide parents with the knowledge of when and how to react to children’s experiences of SR challenge; the second was to improve parents’ knowledge of how to be proactive to shift children from co-regulation (through autonomy-supportive guidance) to (developmentally appropriate) SR. To test the effectiveness of the program, a mixed methods approach was adopted: both parent autonomy-supportive guiding behaviours and children’s SR were measured quantitatively at pre-and post-intervention, and qualitative data gained insight into the feasibility of the program. Specifically, the research was guided by the following research questions and corresponding hypotheses:

**RQ1.**  *Does parent participation in the PiP program have an effect on their autonomy-supportive guiding behaviours?*

**RQ2.**  *Does parent participation in the PiP program have an effect on their children’s self-regulatory performance in everyday play situations?*

It was hypothesised that parents who engaged in the PiP intervention would increase their autonomy-supportive behaviours and their children would show enhancements in their SR abilities more so than parents and children in an active control group.

## 2. Materials and Methods

### 2.1. Design and Participants 

The study was an 8-week randomised led trial (RCT) comparing a parent–child play group intervention (Partners in Play, or PiP) with an active control group. Parents of pre-school children (age 3–5 years) were eligible to participate in the intervention and were recruited through advertising distributed to the Early Start Discovery Space children’s museum member families (~12,000 families), nine local early childhood education and care services, and social media. Participation was compensated by a $20 children’s museum voucher (for admission or gift shop) on study completion. 

An a priori power calculation was conducted using G*Power [58], assuming multiple regression analysis with four predictor variables, a conventional alpha (α = 0.05), power of 0.80 (1-β), and an estimated moderate effect size (f^2^ = 0.25). This yielded a target sample size of 50 parent–child dyads. The randomized control trial was registered with ANZCTR (ACTRN12621001705875).

**Figure 1:**
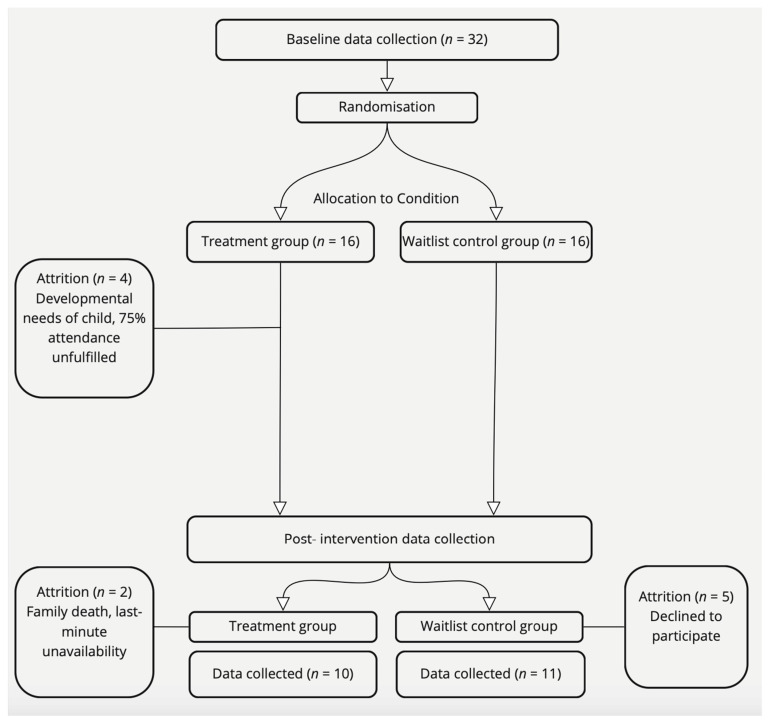
Details of parent–child dyad participation enrolment, attrition, and final number for analysis.

Impacted by the COVID-19 pandemic and fears of escalating infection rates, recruitment efforts yielded a sample of 32 parent–child dyads (30 mothers and 2 fathers). The sample consisted of 16 boys and 16 girls, aged between 3.02 and 5.22 years (*M* = 4.13, SD = 0.60). The flow of participants through the study is shown in Figure 1. Both statistical significance and effect sizes are reported and are considered together in the interpretations and conclusions drawn from the results. However, given that the sample size did not meet recruitment targets—due, for example, to COVID-19 impacts on research—we have given equal privilege to effect size indices.

### 2.2. Participant Randomisation

After baseline data collection, dyads were randomly allocated to either the intervention group or active control group who were waitlisted to receive the intervention. Specifically, participants were allocated a number based on the sequence in which they signed up to the study, and intervention group participants were those whose number was randomly selected by an online random number generator. Randomisation was executed by a co-author who was not involved in intervention delivery or data collection. The remaining participants were allocated to an active control group. Intervention participants were further divided into one of five weekly program session times based on availability.

After randomisation, 11 dyads (34%) did not complete the study. In the intervention group, one child had previously undisclosed atypical developmental needs that precluded their ability to participate in program sessions and, thus, withdrew after the first workshop; three dyads did not fulfil the 75% attendance requirement of workshops due to prolonged illness (influenced and exacerbated by the COVID-19 pandemic); and two dyads completed the program but were unable to attend the post-intervention data collection session due to a family death and last minute unavailability. The remaining five dyads in the control waitlist group declined to participate when invited back for data collection after the intervention period. The final set of dyads (*n* = 21) consisted of 12 girl–mother dyads, 8 boy–mother dyads, and 1 boy–father dyad. Parent demographics are shown in Table 1. The average child age at the beginning of the study was 4.12 years (SD = 0.65). The children who left the study did not differ significantly from the children who completed the study by age (t(30) = −0.08, *p* = 0.937, Cohen’s *d* = 0.61) or sex (as calculated using Fisher’s exact *p* = 0.78). Baseline parent autonomy support did not significantly differ between parents who left the study and parents who remained during structured play (t(30) = 0.40, *p* = 0.692, Cohen’s *d* = 0.81) or during free play (*t*(30) = 0.37 *p* = 0.718, Cohen’s *d* = 0.82). Given randomisation procedures and comparability between groups after randomisation, inclusion of other (e.g., child-level) covariates was deemed non-warranted and, in the context of an already lower-than-target sample size (and thereby lower-than-targeted statistical power), these were not pursued. All analyses were conducted using data from 21 parent–child dyads who completed the study and provided both pre- and post-intervention data. Participant demographic details can be seen in Table 1. 

### 2.3. Intervention

The intervention took place across two sites; in-person components were delivered in a mock pre-school room attached to the Early Start Discovery Space children’s play museum, situated on campus at a university in a metropolitan area of New South Wales, Australia. Components delivered online took place with parents in their homes.

Partners in Play (PiP) is a parent intervention designed based on a prominent model of SR components, influences on SR processes and success, and a Self-Determination Theory (SDT, [32]) and play perspective of how to generate change in these factors. It aims to equip parents with the knowledge, tools, and strategies of knowing when and how to support their children’s self-regulation development during play. SDT characteristics that led to feelings of child autonomy, competence (challenge and encouragement), and relatedness were embedded across three elements: four online parent-first knowledge sessions, each of these followed by a fortnightly on-site play workshop and then at-home play in between the in-person workshops. Across these intervention touchpoints, parents were provided insights, evidence, and coached into how and when to use autonomy-supportive guiding behaviours during parent–child play using the hierarchy of autonomy support [44]. Uniquely for PiP, this was adapted to guide parents in supporting their children in a dyadic play context, offering types of questions and statements such as encouragement, guiding statements, questions, and physical means of support [59]. 

Program design, planning, and implementation placed at the fore the already-present play dynamic between each parent–child dyad that had been cultivated in the 3–5 years prior to this study. Parents’ own autonomy was respected; as such, the intervention did not prescribe specific language or behaviours but rather drew attention to parent behaviours and alternative options with the child at the centre as well as discussing with them (individually) any questions or challenges as they arose. Key tenets of the program can be found in a previously published method protocol [59].

#### 2.3.1. Online Parent-First Knowledge Session

Hosted over Zoom, the online parent-first knowledge sessions aimed to provide parents with the conceptual underpinnings of the program strategies and improve parents’ understanding of child self-regulation and autonomy-supportive guidance. These ran for 45–60 min, once every fortnight at two consistent scheduled times (the second was a repeat of the first to cater for participants’ varied schedules). With permission, the sessions were recorded and shared with participants who were unable to attend either session, prior to the in-person play workshop. The sessions started with a group reflection on the previous week’s practice, during which parents were invited to ask the researcher any questions regarding implementing the strategies in their specific context. Other parents were also invited to share their experience if relevant to the question, thus cultivating a community of practice among the parents. The researcher presented novel information via the screen-share function, after which parents were again invited to ask clarification questions and share their thoughts. 

#### 2.3.2. In-Person Play Workshops

Play workshops were held in a mock pre-school room attached to the children’s museum lasting 90 min, held once every fortnight. Participants attended the same scheduled workshop throughout the entirety of the intervention, with groups comprising no more than 4 parent–child dyads. The workshop began with a 15-min parent-first recap of the online knowledge session (during which a research assistant played with the children), wherein parents could ask the researcher any questions or reflections since the knowledge session. In the remaining 75 min of the workshop, parent–child dyads moved around different play stations of challenging play tasks, while the researcher spent 10–15 min observing and providing each parent with observer insights and suggestions for their autonomy-supportive play practices/strategies (coaching). 

#### 2.3.3. At-Home Play

Following the in-person play workshops and before the next knowledge session, parents were asked to engage in one-on-one play with their child using play resources they already had at home so as to practice and consolidate the principles and strategies from the knowledge sessions and play workshop. Parents’ experiences of this play were reflected upon in subsequent knowledge sessions and served to inform future workshops and individualised support. 

#### 2.3.4. Active Control Group

To match the minimum play dosage of the intervention group, parents in the control group were asked to engage in one-on-one play with their child at home at the same frequency and duration as intervention participants. Parents in both conditions were given a play-at-home calendar to track the time they spent engaged in parent–child play. 

### 2.4. Materials for Data Collection

#### 2.4.1. Child Play Activities

**Figure 2:**
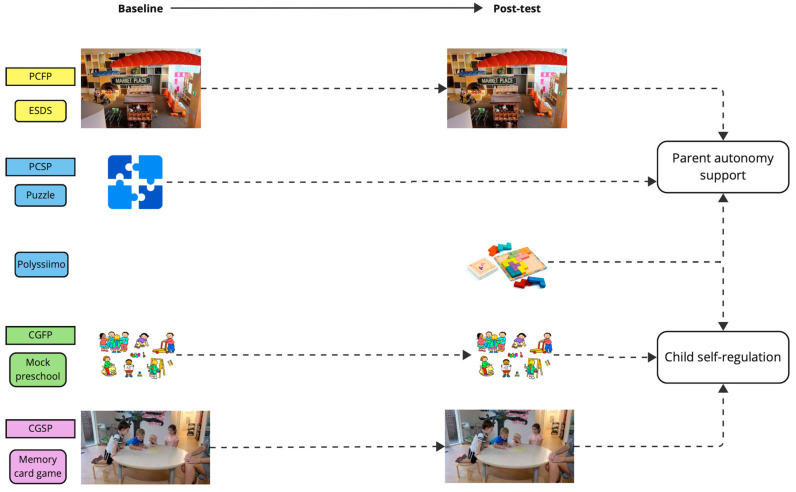
Diagram to show data collection activities, measures, and associated outcomes. PCFP = parent–child free play; PCSP = parent–child structured play; CGFP = child-group free play; CGSP = child-group structured play; ESDS = Early Start Discovery Space.

Data on parent autonomy support and child self-regulation were collected across four key play contexts: parent–child structured play; parent–child free play; child group structured play; and child group free play. These activities, their sequence, and the parent and child measures used for each are depicted in Figure 2. So that the observer could focus exclusively on one particular aspect and measure at a time, parent autonomy support and child SR were largely assessed in different activities (albeit similar in that they were both assessed in structured and free play situations).

##### Parent–Child Free Play (Early Start Discovery Space)

Groups of 3–4 children were first welcomed to the children’s museum and given time for brief free play. Each parent–child dyad was shadowed by a non-participant observer who, at the end of the period, scored the parent on their use of autonomy-supportive behaviours in the free-play context using the adapted Maternal Autonomy Support Scale [60]. This was repeated at post-test.

##### Parent–Child Structured Play (Baseline = Puzzle; Post-Test = Polyssimo)

The parent–child dyads were then each invited into a separate meeting room, where they were asked to complete a puzzle together. A non-participant observer rated the parent on their use of autonomy-supportive behaviours using the Maternal Autonomy Support Scale [61]. At post-test, the activity was replaced with a commercially available puzzle game called Polyssimo. Polyssimo is a pattern completion game consisting of 11 wooden, coloured shapes and a square tiled board. Less and more complex patterns differentiate the difficulty levels of the game, and there is only one possible solution per pattern. 

At post-test only, the observer additionally rated children playing this game using the Preschool Situational Self-Regulation Toolkit (PRSIST [1]), as an opportunity to uniquely consider parent autonomy support and child self-regulation together within the same activity (as all other activities rated one or the other separately). This was added after the start of the program, following the interventionist’s observation that children’s situational SR interacted dynamically with parent autonomy support during in-person workshop sessions. Hence, ratings of child SR in this activity were available at post-test only.

##### Group Structured Play (Memory Card Game) 

Once each dyad had completed both parent–child activities, children convened in a mock pre-school room to play a memory card game in a group of four children, facilitated by the lead researcher. The facilitator–observer rated children’s self-regulatory behaviours using the Preschool Situational Self-Regulation Toolkit (PRSIST [1]). This was repeated at post-test.

##### Group Free Play (Free Play in Mock Pre-School Room)

Upon completion of the memory card game, children engaged in group free play in the same mock pre-school room for 15–20 min. This was video recorded for later scoring using the RRSM [62]. This was repeated at post-test.

### 2.5. Measures

Specific details of each measure are described below and can be found in the previously published method protocol [59]. Inter-rater reliability statistics are also reported below. Table 2 highlights activity and measure employed at pre- and post-test. 

#### 2.5.1. Parent Autonomy Support 

The Maternal Autonomy Support Scale [61] is an observer report scale that consists of four multi-faceted items that assess parents’ contingent responses to a child’s needs (i.e., *Intervene according to the child’s needs and adapts the task to create optimal challenge for the child)*, encouragement given during play (i.e., *Encourage their child in the pursuit of the task, gives useful hints and suggestions, and uses a tone of voice that communicates to the child they are there to help*), parent flexibility during play (i.e., *Take their child’s perspective and demonstrates flexibility in their attempts to keep the child on task),* and parent respect of children’s lead and permits child autonomy (i.e., *follow their child’s pace, provides the child with opportunity to make choices, and ensures that the child plays an active role in the completion of the task).* Parent autonomy-supportive behaviours are scored on each of these items following observation of parent–child play by a trained observer. Each item is scored on a 5-point Likert scale from 1 = not autonomy supportive to 5 = extremely autonomy supportive. The composite autonomy support score generated by averaging these items has shown good validity and reliability (e.g., internal consistency, α = 0.89 [61]. 

The lead researcher and three research assistants underwent training on the use of both the Maternal Autonomy Support Scale [61] and an additional version of the Maternal Autonomy Support Scale (α = 0.88 [60]), which were used in structured play and free play, respectively. Inter-rater reliability was assessed using Fleiss’ kappa coefficient for multiple raters. Raters’ scoring on the Maternal Autonomy Support Scale [61] for structured play achieved a Fleiss’ kappa value of 0.74 across two test instances, which has elsewhere been classified as substantial agreement [63]. A moderate agreement (Fleiss’ kappa = 0.58, [63]) between researchers on one test video was achieved for Matte-Gagné et al.’s [60] adaptation of the measure for the free-play context. The scales were completed in situ while parent–child dyads were concurrently observed by researchers. 

#### 2.5.2. Children’s Self-Regulation

The Preschool Situational Self-Regulation Toolkit (PRSIST [1]) assessment is an observational measure of self-regulation. In this assessment, children’s self-regulation is observed and rated, using an established rating scale, while undertaking a small group (e.g., memory card game) and/or individual activity (e.g., curiosity box guessing game). The tool generates ratings of cognitive self-regulation (for example, *Was the child self-directed, engaging in the activity with little prompting?*) and behavioural self-regulation (for example, *Did the child control their behaviours and stay within the rules of the activity?*). PRSIST has shown good predictive validity [1] and strong reliability (Cronbach’s α ranging from 0.86 to 0.95 [64]. Only the group Memory Card Game from the toolkit was adopted for this study. Video recordings of the children engaging in the activity were later scored by the lead researcher and one research assistant reaching a moderate level of agreement (average Cohen’s kappa score = 0.70). The two scorers completed online training and achieved a correlation or *r* > 0.89.

The Regulated-Related Skills Measure (RRSM, [62]) is an observational tool to measure young children’s employment of Regulation-Related Skills (RRS), described as a combination of skills or processes that draw on children’s self-regulation. Originally designed for the classroom environment, permission was granted from the developers for its use in the free-play context for this study, and support for this use was given by the developing team when necessary. Prior to observation of child play, the mock pre-school room was set up with a variety of toys and activities such as drawing, train tracks, giant building blocks, floor cushions with books, and role play outfits. Children were recorded for approximately 15 min, and their behaviour was later scored by the lead researcher and a research assistant. Both scorers completed RRSM training and achieved moderate agreement between the coding pair (average Cohen’s kappa score = 0.76). 

#### 2.5.3. Parent Perspectives

A parent questionnaire for the intervention group was intended to gather qualitative data in the form of parent perspectives of the PiP program through open-ended questions focused on their perceptions of changes in their own behaviour and that of their children. The questionnaire was also designed to gather information regarding parent perception of intervention implementation characteristics, intended to facilitate researcher understandings of the usability of intervention tools and strategies for parents. 

### 2.6. Procedure

Parents and their children attended baseline data collection, which lasted 90 min. As well as the researcher observing the play, the interaction was also video recorded for quality assurance and second coding if necessary. Some parents chose to leave the room to observe their children through a two-way mirror in a neighbouring room, while other parents stayed in the same room for reassurance but sat at a distance from where the game took place. The end of this play signified the conclusion of the data collection session. Children were invited back to repeat the same data collection session within one week of the 8-week intervention period. The same raters collected data and were blinded to group assignment. Given changes in family availability, group activities were not completed with the same children as at baseline. 

### 2.7. Data Analysis

To evaluate the effect of the PiP intervention, differences between intervention and control of parents’ autonomy-supportive behaviours and children’s self-regulation at post-test, after controlling for pre-test scores and demographic covariates, were analysed through separate hierarchical regressions using IBM SPSS Statistics for Mac (v28.0, Armonk, NY, USA: IBM Corp. 

## 3. Results

### 3.1. Quantitative Data

#### 3.1.1. Initial Data Exploration

Results of an independent-samples *t*-test indicated no significant differences in the age of children between the intervention and active control groups, *t*(19) = −0.89, *p* = 0.386. A Fisher Exact Test confirmed there were no significant differences in the sex of children between the intervention and control group, *p* = 0.081. There was an extreme and plausibly large data point for time engaged in parent–child one-to-one play at home within the active control group, which was trimmed to the next highest non-outlining value. Analyses on the resultant data showed that time spent in one-to-one parent–child play at home did not differ between the intervention (*M* = 111.35 h, SD = 51.13) and active control groups (*M* = 121.13 h, SD = 105.02), *t*(14) = 0.21, *p* = 0.836. The results that follow evaluate intervention efficacy in relation to the two primary research questions and corresponding hypotheses.

##### Does Parent Participation in the PiP Program Have an Effect on Their Autonomy-Supportive Guiding Behaviours? 

Change in parent autonomy support across the intervention period was measured in two distinct play contexts, occurring in the Discovery Space children’s museum and neighbouring rooms: 1—a structured parent-facilitated novel play task; 2—a free-play context. A matrix of correlations between outcome variables is reported in Table A1. Descriptive data of the pre- and post-test scores for each play context are reported in Table A2. In summary, structured-play scores started relatively low in both groups (around 2.5 on a 1–5 scale). In the free-play context, scores started comparatively higher, yet not at the ceiling (between 2.6 and 2.9 on a 1–4 scale). 

A linear regression was conducted to investigate if the grouping variable predicted parent autonomy support post-intervention in each of these play contexts, after controlling for parent autonomy support at pre-test. In the structured-play context, the overall regression was significant (*F*(2, 17) = 15.83, *p* < 0.001, *R*^2^ = 0.65) and there was a large and significant intervention (Group) effect (*β* = 0.81, *p* < 0.001). The average change in the autonomy support score of parents in the intervention group increased from pre- to post-intervention (*M_diff_* = +1.40), whereas the autonomy support score decreased from pre- to post-intervention within the control group (*M_diff_* = −0.36). In the free-play context, the overall regression was not significant (*F*(2, 17) = 1.95, *p* = 0.172, *R*^2^ = 0.19), and there was a non-significant, albeit moderately sized beta for Group (*β* = 0.35, *p* = 0.148). Average autonomy support scores of parents in the intervention group descriptively increased pre- to post-intervention (*M_diff_* = +0.69), compared to parents in the control group whose autonomy-supportive behaviours remained relatively stable (*M_diff_* = −0.04). 

##### Does Parent Participation in the PiP Program Have an Effect on Their Children’s Self-Regulatory Performance in Everyday Play Situations? 

The effect of the intervention on change in children’s self-regulation was also measured in three contexts: structured play (one researcher-facilitated task and another parent-facilitated); group free play; and adult-led transitions in free play. Outcome variables (at baseline and post-test) were evaluated against statistical assumptions for regression. All variables except adult-led transition at post-test were normally distributed (as evidenced by Shapiro–Wilk’s < 0.05, z_skewness_ and z_kurtosis_ < 3); not multicollinear (VIF < 10); and residuals were independent, and similarly and normally distributed (Durbin–Watson ~2, z_residual_ scatterplots, and normal probability plots). No influential cases were identified (all Cook’s Distance < 1). For adult-led transition, data were transformed to normality by reflection and then log10 transformation, which achieved normality (Shapiro–Wilk > 0.05). Results are presented for these transformed data, although we note that both transformed and untransformed versions of the variable produced a non-significant result.

Linear regression analyses paralleled the evaluation of parent effects, although these analyses controlled for child SR at baseline, age, and sex. For the researcher-facilitated structured-play context, results of the overall regression were not significant (*F*(3, 15) = 1.87, *p* = 0.177, *R*^2^ = 0.13), with a non-significant yet moderately sized Group effect (*β* = 0.31, *p* = 0.259). Descriptively (as shown in Table A2, children of parents in the intervention group showed greater growth from pre- to post-intervention on the PRSIST group memory card game (*M_diff_* = +0.55), while children of active control group parents remained relatively stable (*M_diff_* = −0.03). The overall regression predicting PRSIST scores on the parent-facilitated novel task was also non-significant (*F*(5, 14) = 0.918, *p* = 0.498, *R*^2^ = 0.25), with a large but non-significant Group effect (*β* = 0.59, *p* = 0.200). Intervention-group children’s SR performance improved descriptively more from memory card game pre-test scores to novel task post-test scores (*M_diff_* = +1.07) compared to that of the control group (*M_diff_* = +0.52). 

In the free-play context, the overall regression was also non-significant (*F*(4, 12) = 1.86, *p* = 0.182, *R*^2^ = 0.38), with a non-significant and small to null effect of Group (*β* = 0.02, *p* = 0.944). Descriptively, both groups improved their scores from pre- to post-intervention (Intervention group: *M_diff_* = +0.24; Active control group: *M_diff_* = +0.33). The regression on SR scores during adult-led transition was similarly non-significant (*F*(4, 13) = 0.447, *p* = 0.773, *R*^2^ = 0.35) as was the Group effect (*β* = 0.08, *p* = 0.823). The intervention group improved their SR scores (*M_diff_* = +0.37) descriptively (and only slightly) more than the control group (*M_diff_* = +0.09) improved. 

#### 3.1.2. Post-Hoc Explanatory Analyses 

A post-hoc analysis was conducted following an unexpected finding on the Polyssimo task at post-test, which found that, directionally and counter-intuitively, control-group children who experienced lower autonomy support from their parents achieved higher SR scores. To further explore this unexpected finding (in the reverse direction to what we would anticipate), the post-hoc analysis explored child self-regulation and maternal autonomy support correlations within the Polyssimo task, triangulated with assessor field notes during post-test parent–child structured-play activity. This sought to understand the nature (from field notes) and association (by correlations) of parents’ autonomy-supportive guidance with child self-regulation. Results showed that ‘other-regulation’ behaviours were prevalent among the control-group parents, which reflect as lower autonomy support scores despite providing more prescriptive support to children. For instance, parents in the control group would often provide premature support to reduce task demands, prior to children providing cues for help. According to the Maternal Autonomy Support scale, Item 1, for instance, rates parents ‘intervening according to the child’s needs and adapts the task to create an optimal challenge for the child’. While parents’ behaviours were making the task easier for their child, they were not ‘autonomy supportive’ as conceived and operationalised in this research. In this way, parents were reducing the challenge of the task, which did not coincide with a commensurate reduction in child self-regulation ratings. For instance, PRSIST rates whether a child ‘controlled their behaviours and stayed within the rules of the activity’ (Item 5). A requirement for the validity of the self-regulation index generated from this tool is that the activity observed was sufficiently self-regulation demanding. That seemed to not be the case for the active control group children, where the challenge was reduced by the parents.

### 3.2. Qualitative Data

All parents in the intervention condition who completed the questionnaire were mothers (*n* = 9, 90%), with an average age of 36.56 years (SD = 4.64). Eight mothers had 2–4 children, and one was pregnant with her second child at the time of the program. Six mothers participated with their eldest (of two children), while the remaining three children were the youngest (of two). In terms of education level, 66.7% of mothers were educated to an undergraduate degree level; the remaining 33.3% had obtained post-graduate degrees. Six mothers described themselves as Australian, one as Caucasian Australian, one as Anglo Australian, and one as Italian Caucasian. Thematic analysis was conducted in an inductive process: the questions were led by the theory of change and sought to explore if the intervention improved parent knowledge, which led to parent behaviour change and, as a consequence, led to children’s SR change. Yet, the themes that were drawn from the data in answer to these questions were not specifically shaped by any model or framework. 

#### 3.2.1. Understanding Children’s SR

All parents who completed the questionnaire agreed that the program helped them to understand more about their child’s SR. Parent answers were spread evenly across two researcher-constructed themes: improved knowledge and understanding of the concepts on which the intervention was designed and, as a result, the personal reflection of adult behaviour that was specific to the child. Parents were able to utilise their conceptual understanding in reflecting in past behaviour, demonstrating an evolution of practice. 

#### 3.2.2. Improved Conceptual Understanding 

Parents reported that the program improved their knowledge and understanding of SR and were able to apply this new knowledge to their parenting role and commented on a safe workshop environment (*breaking down big concepts, and the iterative cycle of practice, workshop, and feedback*).

Parents recounted that the new knowledge provided them with something through which to view their role with the child’s development as the primary goal, with one parent describing her new knowledge as a ‘lens to wear’. Parents also acknowledged the importance of the parent role toward long-term benefits of early self-regulation development, which they interpreted as a contributing factor toward their behaviour change (as evidenced by the quantitative results). Improved understanding of the concepts that underpinned the intervention content was reported, especially child autonomy and challenge, with one parent saying “*Play that challenges a child, that they need to problem solve and work through frustration and understand how to manage their regulation when they are challenged*” (P10). Another parent articulated her understanding of the importance of support from parents in supporting self-regulation, aligned with the concept of relatedness and competence (in line with SDT [32]) “*The program has helped me to understand the importance of parent participation in the development of self-regulation, the support that parents can give…the need for positive feedback/encouragement*” (P15). Parent responses indicated that the intervention program successfully premised the conceptual underpinnings of strategies that were taught and the rationale behind the intervention, which contributed toward parent investment in modifying their supporting behaviours. 

#### 3.2.3. Personal Reflection and Related Child Behaviour

Not only did parents demonstrate growth in their knowledge and understanding of the concepts within which the PiP program was grounded, but their responses also suggested an improved ability to apply this knowledge during their interactions with their child. This included how they observed their children and also how they responded to children, demonstrating that they could put the conceptual knowledge to action in real-time parent–child play. Respondents felt able to apply the concepts to their parenting behaviour, and through in-depth knowledge of their child, could identify pre-intervention behaviours that were not conducive to child SR growth, with one mother explaining, “*while I believed I wasn’t always ‘doing everything’ for my daughter, I was not giving her enough time or opportunities to complete a task without me intervening…that she most probably did not need* (P24).

Learning about and using the strategies in practice also supported parents in recognising their own strengths in terms of their ability to understand what sorts of circumstances may trigger dysregulation in their child, learning the limits of their child’s capacity to regulate behaviour, and designing the play environment to elicit an optimal level of challenge and autonomy. They were able to operationalise the intervention content to provide individualised support for their child, with one parent reporting “*it gave me an understanding on situations that would trigger her to become unregulated”* (P41). This was echoed by a second parent who identified an improvement in her children’s performance when she “*tried to get both of my kids [2 and 4 years] more involved in the decision making, and this has reduced conflict. I also learned to watch without interfering and have been blown away by how much my children can do, make, negotiate without my help*” (P42). 

Parents reported that participation in PiP supported their learning in how to adapt their guiding behaviours with the child as the focus and, in so doing, optimise conditions for growth. When parents successfully supported their children, this was reported to have a virtuous effect in that their children demonstrated more regulatory behaviours, as evidenced by a parent who noted “*Especially when supported well. And when she successfully regulates, she is super proud and tries again without as much support needed*” (P19). 

#### 3.2.4. Understanding and Actioning How to Support Children’s SR Development 

There was agreement across the parent responses that the program helped them understand how to support their child’s self-regulation, resulting in changes in parent behaviour. Emphasized by seven of the nine parents, the use of the hierarchy of support as a tangible tool to use was essential in putting their new knowledge into action.

1. Hierarchy of Support

The hierarchy of support tool embodied the conceptual core of the intervention and offered parents a practical framework on how and where to pitch support for their child. Along with knowledge gained through learning about individual trigger points to dysregulation, parents were able to give guidance to sustain but successfully tackle challenge at moments of need, contingent on the child’s cues yet nurturing the child’s autonomy in problem solving: 

Respects autonomy and provides gentle prompts to encourage children to self-regulate—it has encouraged me to explore the ‘middle ground’ when intervening i.e., I don’t have to do nothing or do everything, there is lots of in-between support options (P40). 

In action, parents reported that, with practice, they gained confidence in exposing children to challenge, waiting for child cues, and understanding their child’s capacity for challenge. One parent articulated these points and the iterative implementation of the tool that followed: 

I no longer give or try hard not to give her any scaffolding or the answer to a problem she has straight away. I have learnt to offer encouragement first. I am also better at reading when she appreciates this encouragement and when I actually need to offer support in some degree before she has a mini meltdown or is likely to give up (P24).

Another parent described the intervention as giving “’permission’ for caregivers to wait and observe rather than jump straight to intervention, which I have seen as being great for building autonomy and skill use” (P40). This suggests that the parents had confidence in the concepts and theories that underpinned the strategies designed for the intervention and trusted their use of intervention tools.

One parent went into detail about a specific activity with her daughter where she used the program strategies and tools during at-home play. The reflection details not only the parent’s strength in her ability to identify her behaviour change but also the direct influence on her daughter’s problem solving and the parent’s awareness of contextual factors that limited her capacity for regulating behaviours (tiredness).

Prior to this program I would have opened it [LEGO set] with her […] and gone through it step by step. If she got a piece wrong, I would have told her straight away and tried to help her fix it. However, because of this program and what I have learnt, I cleared the kitchen table and told her to go for it […] She had to open the box herself, get the scissors herself to open the plastic (all things I would have already done for her previously). […] We still came across many hurdles, I just used the strategies that [researcher] had taught me. “Does that look like the picture?”, “Is there another way you could do it?”, “Remember when you made the LEGO farm scene following the instructions” positive, encouraging language. I didn’t tell her when she had put pieces in the wrong spot, and it was really interesting to see when she realised that she had. You could see her thinking, trying to work out what she had done wrong. But it just goes to show how many times I have stepped in and solved her problems before she has asked me to (P24).

2. Play Adaption

Parents reported that they adapted the play (in both content and context) to incorporate self-regulation promoting strategies that were taught throughout PiP, signifying that parents saw value in what they were learning, acknowledging the importance of parent–child play. One mother explained that she adapted her role in play “to more consciously steer play as a mechanism for supporting growth.” (P29). During her workshop reflections, the same mother described this in more depth in the context of role play: 

I have realised in order to adopt this framework I don’t have to change the way we play to a structured activity. Simply having the awareness of this framework and organically integrating it into play preferences led by [child] just gives me the opportunity to extend the learning, and actually makes the play more interesting for me to engage in as well as her”. 

This parent quote illustrates parent–child play as a social act in which play can incorporate learning and joy. This parent was able to utilise the strategies within the play that her and her child most commonly engaged in, thus illustrating the individualised nature of the intervention. 

Furthermore, parents reported knowing how the environment can elicit challenge in play (through planning play and increasing freedom in play) and pausing play for continuity over time. The intervention thus afforded parents with the tools to follow the child’s pace during play to support SR but also to nurture child autonomy and long-term SR through choice and continuity of play influencing both ‘bottom-up’ and ‘top-down’ influences toward SR growth: “[child] now accepts ‘pausing’ the play and coming back to it (before I would rapidly take over to get it finished so we could pack it away) and I am slowly using my words more than my actions to guide her.” (P20).

3. Extending PiP

Responses illustrate both the flexibility of the intervention strategies in their use across contexts and, thus, the perceived significance of the program by parents. Outside the participant dyad, wider family members started to use the tools, and in one case a parent translated the hierarchy of support to her professional (formal education) context, demonstrating a clear integration of the intervention concepts and principles in both her home and professional life. PiP offers a framework for parents to better observe and understand children’s needs, resulting in a range of development areas across the participant cohort. One parent noticed a clear difference when playing games, explaining “[child] is better able to follow the rules, and less likely to abandon the game when things do not go his way/he is not winning.” (P15). Even though the strategies were not designed for a tailored emotional regulation intervention, the program supported the parent to better understand her child and his cues for help in a supported framework, which translated to different instances where his regulation was challenged.

4. Changes in Child’s Behaviour

Parents were asked to describe any differences or changes they observed in their child’s behaviour as a result of their participation in the program. Across the data set, one overarching theme was generated: independent problem solving with metacognition.

All but one parent described their child as more able to demonstrate independent flexible problem solving as a result of participation in PiP. Some also mentioned that their child would feel a sense of pride, aligned with feelings of competence, as per SDT [32]. One parent recounted how her child learnt the implicit rules of guidance: “she will ask me for help but then finish my sentence for me, “I know, have a go first.” (P24). This child also changed her questions to ask for help as opposed to asking her parent to fix the problem in the first instance “Previously it would have been “Mum, can YOU do this, can YOU help me…can YOU, can YOU”. These reflections show how quickly children adapted to their parents’ change in support; after the 8-week intervention period, children were able to verbally express their actions and adapted their own help-seeking language to align with the parent autonomy-supportive guidance offered. 

### 3.3. Interpretive Summary

The questionnaire asked parents to reflect on how the PiP program impacted their conceptual knowledge and, thus, understanding of their own child’s SR, their understanding of how to support their child’s SR development, their perceived changes in their own behaviour, and their perceived changes in their child’s behaviour. Parent responses indicated that participation in the PiP intervention had a positive impact. The conceptual basis of the program (delivered through parent-only knowledge sessions and reinforced through coaching and reflection conversations) supported parents to view their guiding behaviours through a new lens and to better observe their child’s regulatory behaviour via everyday manifestations of SR. This improved understanding gave parents a firm basis on which to implement autonomy-supportive strategies through the hierarchy of support tools but also in becoming more comfortable in observing their child’s cues for support and allowing their child to experience challenge. Parents shaped children’s play preferences to maximise opportunities for SR development. This included not only in-the-moment contingent support but also how to shape the play environment to cultivate opportunities for exposure to challenges toward long-term SR growth. In response, parents reported that their children demonstrated more regulation-related behaviours such as persistence, emotional regulation, play continuity, and planning.

## 4. Discussion

We found that parent participation in the PiP program had a significant effect on parent autonomy support in structured play such that intervention group parents increased their use of autonomy-supportive behaviours compared to control group parents. We found that the intervention was not shown to have a significant impact on child SR in structured play in a memory card game, although the direction and size of effects (in light of the child sample size) were in the expected direction. 

Parents who took part in the intervention program were able to adopt more autonomy-supportive guiding behaviours characterised by providing choice, respecting child pace, and taking the child’s perspective when faced with events that challenge the child’s SR. This improvement was not a function of more time or experience engaged in one-on-one parent–child play, because the active control group also engaged in dyadic play, but rather the behaviour change was specific to the play support provided by the intervention. Supported by qualitative data, parent responses emphasised the intervention’s role in improving conceptual knowledge that led to strategy implementation, from which parents recognised changes in both their own and their child’s behaviour. While the efficaciousness of this current intervention evaluation was primarily grounded in quantitative data, which demonstrated positive changes in study outcomes, this was supplemented with self-report questionnaires completed by parents. Responses echoed the effectiveness of the program with parents reporting enhanced knowledge, better capability to observe children’s regulatory behaviours, and the successful use of program tools. Caution is warranted, however, given the potential for bias from unblinded participants as sources of data for primary outcomes. Indeed, self-report measures and large effects are seen by those more willing to engage in the intervention (as found in similar educator-based SR programs [37]. Nevertheless, triangulation with the quantitative results from this RCT suggest that participation in PiP did generate positive parent behaviour change in ways that are conducive to child SR growth. Importantly, as characterized by autonomy-supportive guidance, the intervention taught parents to follow child cues for help and in so doing allowed them to be exposed to SR challenge. Grounded in SDT [32] and supported by meta-analyses [65] and systematic reviews [10], guiding behaviours that allow children to experience autonomy, challenge, and provide contingent positive feedback promote higher SR behaviours. 

PiP is unique to other SR interventions given that it targeted parent change rather than educator change. Research tells us it is what parents do, not who parents are, that matters the most with regard to experiences afforded to children and the associated cognitive attainment [13]. The PiP program offers a possible solution to providing parents with the tools to offer their children more quality playful learning experiences that bring about cognitive growth. It is largely accepted that play is essential toward children’s physical, social, emotional, and cognitive development, yet there has been a worrying decline in the opportunities of play for many children [66,67]. While family play is highly beneficial for both adult and child well-being (see [68]) the current study has shown that capitalising on parent–child play time can further enhance children’s self-regulation development. While SDT theory posits relatedness as necessary for intrinsic motivation to regulate behaviour toward a goal, our study extends this view, and while play may offer an ideal opportunity for parents to fully engage with their children [66], it is not simply a case of the amount of family play that benefits children’s cognitive development but rather the quality of the play interaction. With long-standing evidence suggesting the significance of the HLE, the experiences offered by parents impact greatly on children’s development. In cultivating an environment of security and genuine care from which children are likely to take on challenges and exploration, parents have the upper hand over educators. 

The provision of feedback has been shown to result in greater SR change [10], and the encouraging language used in the PiP tool praised children’s effort, encompassing open-ended questions [69] and suggestions that helped children identify mistakes themselves, which also contribute to children’s self-regulation skills [41,42,43,45,70]. Such feedback leads to a mastery approach to challenging tasks that also contributes toward regulatory behaviours [71,72]. Not only does this language satisfy the feedback component of competence in SDT, but it also serves to fulfil a child’s need for autonomy [73,74,75] via contingent parent input. Neale and Whitebread [76] found a reciprocal, positive feedback loop between contingent responses that led to child success and child success that led to contingent responses: “contingency is a means of maintaining a child’s goal-directed focus, while continually emphasising the importance of their personal agency in achieving that goal” (p. 269). The intervention enabled parents with the tools and knowledge for not only how but also when to intervene: a critical element for successful and sustainable guidance. 

Although we found child SR change in the hypothesised direction, the non-significant result requires consideration. The null effects may, for instance, be a product of the low-risk sample, highlighted by the homogeneity of the parent participants in socioeconomic status and education. Interventions are often most effective for at-risk children who initially achieve lower SR scores [36,77,78]. Also, the smaller-than-target child sample size caused lower than anticipated statistical power and, thus, limits the chance of identifying any effects to only those quite large in size. There are other context-dependent factors that may have also impacted the results: the 8-week duration period may not have been long enough to embed consistent child changes with this sample. This is especially relevant when considering busy work–childcare schedules and multiple-child families—raising challenges for dedicating time for one-on-one parent–child play. The intervention duration may have benefitted from being longer in order to sustain the practice over time, during which children may have encountered more and varied instances of SR challenge for parents to practice the program strategies across a range of scenarios, which may also counteract the unstable nature of SR and offer more opportunity for parents to support children with triggers that impact reactivity to in situ SR challenges. Duration and dosage both remain conflated issues in intervention research, however, with no clarity in their association with effective SR change [39]. 

Given the low statistical power, we gave equal consideration to effect sizes, which were calculated using http://www.psychometrica.de/effect_size.html accessed on 9 April 2021. Effect sizes for parent behaviour change position the PiP program (*d* = 1.91) as highly effective, when compared to those included in a meta-analysis of sensitivity and attachment interventions in early childhood (for which parent autonomy support scores are available [38]. Bakermans-Kranenberg et al. [38] found that the aggregate Cohen’s *d* effect size for the 51 RCT interventions included was *d* = 0.33, it was and *d* = 0.38 for those consisting of 5–16 sessions (as did the current program). Effect sizes for child SR in structured play (*d* = 0.48) also position this amongst the more effective interventions compared to those included in a recent review of SR interventions [10], which found effect sizes ranging from Cohen’s *d* 0.10 to 0.43. For free play and adult-led transitions, effect sizes in the current study situate these elements of the program as within (but at the lower end of) effects generated by previous interventions. 

A surprising and counter-intuitive finding, explored via post-hoc analysis, was a negative association for control group parent autonomy support with children’s SR at post-intervention in the Polyssimo game. In this case, prescriptive parental guidance led to seemingly higher child SR scores, characterised by a directedness to solutions. With reference to the nature of parents’ autonomy-supportive behaviours, results showed that ‘other-regulation’ behaviours were more prevalent among the control-group parents, which reflected as lower autonomy support scores through providing more prescriptive support to children. Prior to children’s cues for help, parents in the control group provided support prematurely that reduced task demands and were therefore not ‘autonomy supportive’ as operationalised by either the autonomy support measure used or the conceptualisation of autonomy support for this research. 

Cook and Artino [33] posit that the competence component of SDT is cultivated through optimal challenge and positive feedback. While the literature is clear on the importance of challenge for cognitive growth [79], prescriptive parental guidance results in the reduction or removal of a challenge such that children need not adopt regulatory behaviours at all since their distracting impulses are overridden by parents, as shown by the control-group parents in the current study; by removing the challenge for their children, they may have ‘other’-regulated them by making the task easier.

The second surprising finding was that we did not find intervention effects for parent autonomy support nor children’s SR in free play. The lack of intervention impact in the free-play context supports the notion that free play is inherently self-regulatory [50], supported by qualitative data from parents who reported that their children rarely requested help when they engaged in free play at home. Free play can be characterised by the presence of choice and flexibility, for personal enjoyment, and as being self-motivated [80], where children, through the use of cultural tools [48], “’build’ internal representations which guide their behaviour” [50], p. 1696. The literature refers to a range of different activities that fall under the umbrella term of free play (for example, role play, imaginative play, symbolic play, and creative/artistic play) with one common element—they are self-paced [81]. Key principles of autonomy-supportive behaviour used in PiP prioritise children’s pace through choice, contingent support, and relevant feedback; thus, it could be suggested that autonomy-supportive guiding strategies support SR development because they help to establish a play environment that mimics, by virtue of the strategies in place, the self-paced element of free play: the child owns, directs, plays at their own pace, and problem solves in the (relative) absence of adult directives. Through autonomy-supported structured play, i.e., self-paced play, children are afforded experiences to help develop, utilise, and demonstrate growing SR capabilities. 

### Limitations and Future Research

Despite promising findings, this study is not without its limitations. Measuring self-regulation offers its challenges, not least due to the conceptualisation in which it is thought of here. By design, the play activities were structured as challenging tasks; therefore, they required cognitive and associated behavioural regulation more so than social–emotional. The design of the tasks and the accompanying supportive strategies, while engineered to elicit challenge and support children through challenge, were perhaps not conducive to nurturing overall SR. The unstable nature of SR, influenced by contextual factors, was also evidenced and highlights questions surrounding how we measure the ‘bottom-up’ facets of a child’s regulatory abilities. If SR is unstable across the same context, how do we account for its fluctuations that are so heavily dependent on factors outside of experimental control? Indeed, the aim of this study was to optimise everyday play activities for SR growth, but for a child, with every day comes contextual changes that impact their ability to regulate. While this does not affect strategy implementation, it does impact rigour in relation to measuring SR in a controlled way. 

The sample was homogenous in terms of high levels of education and advantage. While home resources were not captured, the socioeconomic indices for the areas in which the children live are almost all high. It is expected, however, that program effects should still generate across contexts. Albeit evaluated with highly educated parents, the intervention was not designed for parents with a particular level of education. Design elements such as the face-to-face workshops and coaching ensure that parents received sustained support throughout the program, so the program can be responsive to the needs of the participants. Parents are also given tools and strategies to use in the home environment rather than a set of prescriptive activities that require specific play equipment. Interventions tend to show that those who gain the most are those that have the most to gain [82]. Given that strong effects in the current study were found in children who, by virtue of their demographics, we might expect to see less positive change, it stands to reason that stronger effects may be found if the program were used with less-advantaged groups. While the current data cannot speak to these possibilities, and future research is required, this study provides a basis for implementation with groups other than affluent parents who opted for participation in this instance. 

The study demonstrated an efficient and sustainable way to educate parents in supporting their child’s SR outside of the formal learning context. The University’s mock pre-school space offered a hub in which groups of parents could gather in a community of practice with the researcher, as opposed to the researcher engaging in home visits, parent by parent. Appealing to a wider audience and demographic—with the inclusion of more fathers—would shine a light on the potential scope of the intervention for all parents, irrespective of demographic or education level, to learn the conceptual underpinnings of the intervention and apply it to their everyday play practices with their children. One possibility of expanding beyond the context in which the study was conceived is to engage pre-school and educational practitioners as facilitators, adopting a pyramid method of intervention. 

The challenging COVID-19 context through which this study was implemented potentially negatively influenced initial recruitment numbers. Study recruitment commenced during a period of emerging cases and restricted university-based research procedures, impeding participant willingness and engagement. The program was implemented in a 2-month period directly preceding a nationwide lockdown in June 2021. Parent fear and stress related to the pandemic led to an increase in controlling parent behaviours and heightened perceived environmental stress [83]. Growing community concerns in the lead up to project completion are likely to have influenced higher than anticipated attrition rates. The control waitlist did not receive the intervention due to state-wide travel restrictions and University COVID-19 measures that rendered human face-to-face research impossible. Instead, these participants were sent an infographic (under construction) detailing the intervention strategies. 

## 5. Conclusions

The Partners in Play (PiP) intervention was conceptualised in response to a paucity of SR interventions in the HLE and associated contexts. PiP taught parents when and how to support children through the use of autonomy-supportive guiding strategies, resulting in changes that aimed to nurture children’s SR development. The study demonstrates that parents’ use of autonomy-supportive strategies, which are contingent on the child’s cues for help, can encourage children to learn how to work through cognitive and self-regulatory challenges. This was not parents’ natural inclination, as demonstrated in baseline autonomy support data. While exact mechanisms would need to be further evaluated, the theory of change underpinning PiP proposes that support for children’s authentic, in situ reactivity and approaches to SR challenge supports both contextual (bottom-up) and capacity (top-down) influences on SR growth. This intervention proposes a promising strategy for sustained SR improvement over time, particularly in its ability to leverage the practices of parents—who are children’s first, most important, and most influential teachers.

## Figures and Tables

**Table 1:** Participant demographic information.

	Intervention	Control
Parents		
*n*	9 (9 mothers)	9 (8 mothers, 1 father)
Age in years, M (SD)	36.65 (4.64)	38.33 (4.50)
Number of children	1 had 1 child (pregnant with 2nd)	1 had 1 child
	8 had 2–4 children	8 had 2–4 children
	1 did not report	2 did not report
Education	0 TAFE (college)	1 TAFE (college)
	6 undergraduate degree	3 undergraduate degree
	3 post-graduate degree	5 post-graduate degree
	1 did not report	2 did not report
IRSAD (SES) quintile	1 in quintile 1	1 in quintile 1
	5 in quintile 4	5 in quintile 4
	3 in quintile 5	3 in quintile 5
	1 did not report	2 did not report
Children		
*n*	10	11
% female	72	36
Age in years, M (SD)	4.25 (0.71)	4.00 (0.59)

TAFE = Technical and Further Education (roughly equivalent to college). IRSAD (Index of Relative Socio-Economic Advantage and Disadvantage) is an area-level index of SES drawn from the 2016 Australian Census. Reported here is IRSAD quintiles from 1 = most disadvantaged to 5 = most advantaged. Demographic survey return rates were 90% of intervention-group parents and 82% for control-group parents.

**Table 2:** Activity and measure employed at pre- and post-test.

Child Activity	Play Activity	Pre-Test	Post-Test
Parent–child structured play	Puzzle (T1) Polyssimo (T2)	Maternal Autonomy support scale	Maternal Autonomy support scale and PRSIST
Parent–child free play	Discovery Space	Maternal autonomy support scale (adapted)	Maternal autonomy support scale (adapted)
Child group structured play	Memory card game	PRSIST	PRSIST
Child group free play	Mock pre-school	RRSM	RRSM
Adult-led transition	Mock pre-school	RRSM	RRSM

## Data Availability

The data presented in this study are available on request from the corresponding author due to ethics approval protocols.

## Appendix A

**Table A1:** Correlation matrix for study variables.

Variable	1	2	3	4	5	6	7	8	9	10	11
1. Age	-	0.42	−0.27	−0.04	−0.19	−0.08	0.40	−0.56 *	0.46 *	0.42	−0.16
2. SR Structured T1		-	0.07	0.50 *	−0.01	−0.03	0.78 **	−0.15	0.24	0.31	0.23
3. SR Free Play T1			-	0.41	−0.35	−0.04	−0.13	0.44	−0.50 *	−0.28	0.23
4. Transition T1				-	0.21	0.31	0.14	0.20	0.11	−0.24	0.27
5. AS Free Play T1					-	0.25	−0.11	−0.05	0.28	−0.23	−0.11
6. AS Structured T1						-	−0.26	0.09	0.25	−0.02	−0.35
7. SR Structured T2							-	−0.46 *	0.34	0.45	0.45
8. SR Free Play T2								-	−0.43	−0.42	0.02
9. AS Free Play T2									-	0.48 *	−0.07
10. AS Structured T2										-	−0.15
11. Transition T2											-

SR = self-regulation. AS = parent autonomy support. T1 = baseline. T2 = post-intervention. * Correlation is significant at the *p* < 0.05 level. ** Correlation is significant at the *p* < 0.01 level.

## Appendix B

**Table A2:** Descriptive table detailing the mean scores of the study outcome measures at pre- and post-intervention.

	Intervention					Control				
Outcome Measure	Pre M	SD	Post M	SD	*Mdiff*	Pre M	SD	Post M	SD	*Mdiff*
Maternal Autonomy Support										
Structured play	2.44	0.95	3.83	0.75	+1.40	2.50	0.82	2.13	0.57	−0.37
Free play	2.62	0.50	3.31	1.11	+0.69	2.92	0.61	2.89	0.84	−0.03
Children’s SR										
PRSIST										
Memory card task	4.11	1.11	4.66	0.83	+0.55	3.75	1.22	3.72	1.19	−0.03
Polyssimo (T2 only) *	-	-	5.17 *	1.14	+1.06	-	-	4.27 *	1.26	+0.52
RRSM										
Free play	3.24	0.47	3.48	0.32	+0.24	3.30	0.63	3.63	0.41	+0.33
Transition	2.56	0.83	2.93	0.68	+0.37	2.99	0.74	3.07	0.70	+0.08

Note. * Denotes child SR scores on the additional Polyssimo game, which was administered at post-test. There are minor discrepancies between inferential and descriptive results between the table and text due to rounding in the analytical software. For the maternal autonomy support scale, structured play scores can range from 1 to 5, and free play scores range from 1 to 4. PRSIST scores can range from 1 to 7 and RRSM scores can range from 1 to 4 (where 1 and 2 are variations of when the child ‘does not’ and 3 and 4 are variations of when the child ‘does’).
